# Improvement of Trehalose Production by Immobilized Trehalose Synthase from *Thermus thermophilus* HB27

**DOI:** 10.3390/molecules23051087

**Published:** 2018-05-04

**Authors:** Jing Sun, Shizeng Wang, Wenna Li, Ruimin Li, Sheng Chen, Hyon Il Ri, Tae Mun Kim, Myong Su Kang, Lu Sun, Xinxiao Sun, Qipeng Yuan

**Affiliations:** 1 State Key Laboratory of Chemical Resource Engineering, Beijing University of Chemical Technology, Beijing 100029, China; wdboxyx@126.com (J.S.); shizengwang@gmail.com (S.W.); 13426481082@163.com (W.L.); 15652908349@163.com (R.L.); chensheng320@126.com (S.C.); 2 Department of Chemical Science, KIM HYONG JIK University of Education, Pyongyang 999093, Democratic People’s Republic of Korea; rhlbuct@163.com; 3 Department of Chemical Science, KIM IL SUNG University, Pyongyang 999093, Democratic People’s Republic of Korea; 2013410010@mail.buct.edu.cn (T.M.K.); tbigdragon@126.com (M.S.K.); 4 Research Center of Futaste Pharmaceutical Co. Ltd., Yucheng 251200, China; biosyn2016@126.com

**Keywords:** enzyme immobilization, trehalose synthase, reusability, stability, silicalite-1

## Abstract

Trehalose is a non-reducing disaccharide with a wide range of applications in the fields of food, cosmetics, and pharmaceuticals. In this study, trehalose synthase derived from *Thermus thermophilus* HB27 (TtTreS) was immobilized on silicalite-1-based material for trehalose production. The activity and the stability of TtTreS against pH and temperature were significantly improved by immobilization. Enzyme immobilization also led to a lower concentration of byproduct glucose, which reduces byproduct inhibition of TtTreS. The immobilized TtTreS still retained 81% of its initial trehalose yield after 22 cycles of enzymatic reactions. The immobilized TtTreS exhibited high operational stability and remarkable reusability, indicating that it is promising for industrial applications.

## 1. Introduction

Trehalose is a non-reducing disaccharide consisting of two glucose units linked by α,α-1,1-glycosidic linkage [1]. In nature, trehalose can be synthesized by insects, microbes, plants, and mammals to protect subcellular structures against osmotic stress, refrigeration, dehydration, high temperature, and other harsh environments [2,3,4]. These chemical and biological properties render trehalose a wide range of applications in the food, cosmetics, and pharmaceutical fields, ranging from a sweetener to a biomaterial stabilizer [5]. Recently, it was reported that trehalose also showed applications in the treatment of Alzheimer’s disease by triggering autophagy [6].

Trehalose can be produced by trehalose synthase (TreS) from maltose [7]. TreS is efficient, practical, and low-cost for the industrial production of trehalose, since it involves only one step to convert maltose into trehalose by intramolecular rearrangement in vitro [8,9]. Recently, TreS derived from *Thermus thermophiles* (TtTreS) was successfully expressed in *Escherichia coli* to produce trehalose [10]. Our recent research indicated that the activity of TtTreS could be further improved by adding the C-terminal domain [11].

During trehalose production, one of the obstacles is the poor stability of TreS under reaction conditions. The activity of TreS was also limited by temperature and pH. Compared to immobilized enzymes, soluble enzymes in mixture tend to form aggregates, which may alter their activity and stability. Researchers suggest soluble enzymes may not be adequate for industrial production [12]. Immobilization is an effective method to improve the stability and reusability of free enzyme. Materials are important for immobilization and they have been the subject of considerable investigation [12,13,14,15]. Mechanical properties, particle size, activity, selectivity, inhibitors, relevance of the support pore size, and other elements should be considered in immobilization [12]. For example, considering pressure problems and so on, the mechanical properties are closely connected to the setup of the reactor [16]. Regarding substrate diffusion and consumption of biocatalysts, although it seemed that large column-shaped particles are more favorable than small ones, they are subject to diffusional problems [12]. Immobilization affects the enzyme activity and produces distortions which may alter enzymatic properties [17]. Generally, larger pores bring smaller specific area [12]. To select an appropriate immobilization method, the catalytic process, enzymatic properties, the choice of supports, and other elements should be considered [18].

It was reported that silanized magnetic ferrous-ferric oxide, Eupergit C250L, and polystyrene divinylbezene-based metal chelators could be used for TreS immobilization [19,20,21]. After the conditions were optimized, an immobilized system of trehalose synthase using Eupergit C250L reached a 42% yield of trehalose [20]. Many methods are used for immobilization, such as using pre-existing supports via reversible physical protocols or the irreversible covalent coupling method [16]. Covalent binding is the method we used for immobilization. This irreversible method proved to be efficient in enhancing the properties of enzymes. In this study, silicalite-1, which was an inexpensive and synthetic material with porous structure and large specific surface, was used as supporting material for immobilization. The hydrophilic surface of silicalite-1 provides the possibility for biochemical reaction with enzymes or other groups [22]. In addition, silicalite-1 with immobilized trehalose synthase is easier to be separated from the mixture than free enzymes by filtration or other methods.

In the present study, we are reporting the continuation of our former report describing trehalose production by recombinant TtTreS [10]. In this study, we used a 2.5 L fermenter to scale up the production of recombinant TtTreS. The crude TtTreS was immobilized on the surface of glutaraldehyde-3-aminopropyltriethoxysilane-silicalite-1 (GA-APS-silicalite-1, silicalite-1 modified sequentially with 3-aminopropyltriethoxysilane and glutaraldehyde) without enzyme purification. In this case, a nucleophilic attack took place at the aldehyde group of glutaraldehyde by the amino group of the protein to form a Schiff base [23]. The activity and stability of immobilized TtTreS against pH and temperature was evaluated by activity analysis. TtTreS after immobilization retained high trehalose yield after 22 repeated cycles and reduced inhibition of TtTreS caused by glucose. Also, the immobilized TtTreS exhibited high operational stability and remarkable reusability without enzyme purification which would increase the cost of production, indicating that immobilized TtTreS is promising for industrial applications.

## 2. Results and Discussion

### 2.1. Immobilization and Characterization of TtTreS on Silicalite-1-Based Support

The production of TtTreS was carried out by a 2.5-L fermenter. By the end of fermentation (25 h), the OD_600_ of *E. coli* and TtTreS activity of fermentation broth reached 40 and 24,666 U/L, respectively. The fermentation time was decreased to 25 h from 30 h, which was needed for fermentation in flasks [10]. The TtTreS activity of unit fermentation broth was also improved after fermentation scale-up by fermenter. Enzyme immobilization allowed not only an increase in stability, but also a better control of the reaction, a higher flexibility for reactor design, and easiness of enzyme recovery and reutilization compared with free enzyme [24]. For immobilization, the preparation of enzyme is reported to play an important role in stability of enzymes. Enzyme loading, immobilization rate, and other factors exhibited positive effects on the final properties of the enzyme, but sometimes they showed negative effects [25]. In our study, in order to obtain a relative high loading capacity, optimizations (GA concentration, protein dosage, and immobilization time) were made to immobilize TtTreS. The best relative activity reached in our study was under GA concentration of 0.25% (*w*/*w*). The effect of protein dosage on the relative activity of immobilized TreS was analyzed, and the best ratio of the enzyme dosage and the amount of the support was 20 U/g.

**Figure 1:**
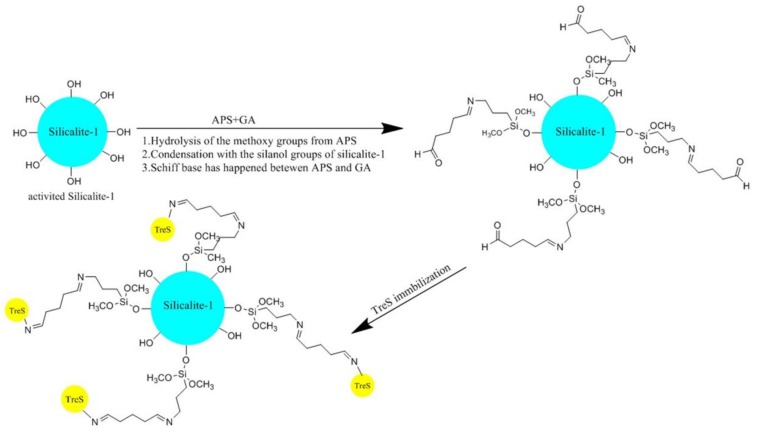
Immobilization process of TtTreS (trehalose synthase (Tres) derived from *Thermus thermophilus* HB27) on silicalite-1-based material.

**Figure 2:**
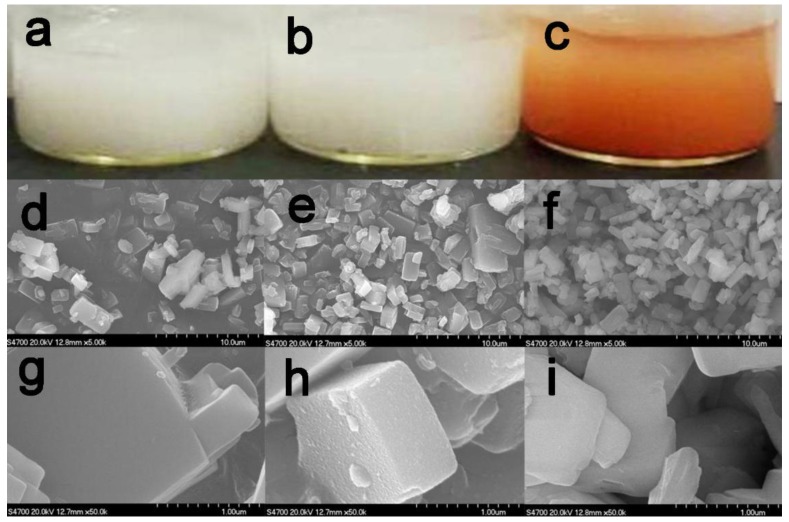
Images of three silicalite-1-based materials. The color appearances of activated silicalite-1 (**a**); APS-silicalite-1 (**b**); and GA-APS-silicalite-1 (**c**); the SEM image of activated silicalite-1 (**d**); APS-silicalite-1 (**e**); and GA-APS-silicalite-1 (**f**) with scale bar of 10 um; the SEM image of activated silicalite-1 (**g**); APS-silicalite-1 (**h**); and GA -APS-silicalite-1 (**i**) with scale bar of 1 um.

The support materials of activated silicalite-1, APS-silicalite-1, and GA-APS-silicalite-1 were prepared sequentially as shown in Figure 1. The color appearances and SEM images of the three materials are shown in Figure 2. The red color of GA-APS-silicalite-1 indicated that the carbon=nitrogen double bond (Schiff base) was formed between the aldehyde groups of GA and the amino group of APS (Figure 2c) [26]. In the present study, TtTreS was immobilized by the formation of a Schiff base between the aldehyde groups of GA and the amino group of TtTreS. The immobilization effect of three silicalite-1-based materials was evaluated by enzymatic reaction at optimal conditions (Table 1). When using activated silicalite-1 and APS-silicalite-1 as immobilization support, the complex of TtTreS and support completely lost activity in the second batch of trehalose conversion, indicating that the TtTreS on activated silicalite-1 and APS-silicalite-1 was possibly immobilized by adsorption. The immobilization by adsorption was unstable and not suitable for TtTreS recycling. However, for the TtTreS immobilized on GA-APS-silicalite-1, trehalose yield (60.28%) in the second batch still retained 98% of that (61.52%) in the first batch, further confirming that TtTreS was successfully immobilized on GA-APS-silicalite-1 by covalent bonding rather than adsorption.

Researchers have reported great work on the operational stability of enzymes. For example, alcoholysis reactions are limited in their mass transfer effects. The group of researchers compared different modified derivatives of a material and found all the chemical derivatizations used in this experiment improved the resistance to rupture [27]. Glutaraldehyde was used in our study as crosslinker. Many aspects of crosslinking glutaraldehyde were studied for their importance in bioconversion [23]. Immobilized enzymes on pre-activated supports of glutaraldehyde were tested. The results showed that the supports with glutaraldehyde improved the operational and storage stability. Being bonded with *Candida rugosa* B (a lipase), the activated support was found to have improved operational stability and performance [28].

Glucose is a byproduct of TreS in the reaction of trehalose conversion [29]. Glucose can dramatically inhibit the catalytic formation of trehalose, suggesting that a decreased concentration of glucose may increase the production of trehalose [29]. In this study, the results showed that the immobilization of TtTreS decreased the glucose concentration of the mixture from 22 g/L to 15 g/L, which could improve the efficiency of the enzymatic reaction. In previous reports, researchers found immobilization could reduce the inhibition of reactions [16]. The immobilization of enzymes may cause changes in enzyme structure, such as blocking the inhibition site [12,16]. The reduction of glucose might be attributable to TtTres being a multimeric enzyme. 

### 2.2. Effects of Temperature and pH on the Activity of Free and Immobilized TtTreS

**Figure 3:**
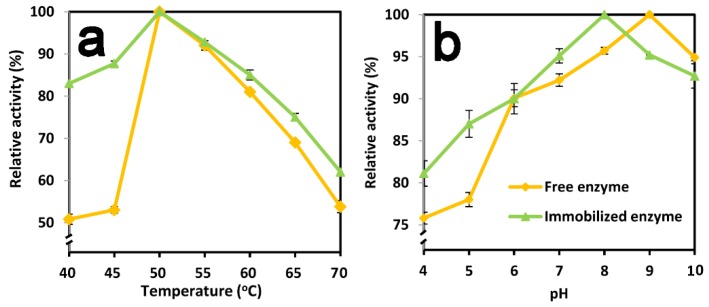
Effect of temperature and pH on the relative activity of free and immobilized TtTreS. Effect of temperature (**a**), and effect of pH (**b**).

Enzyme performance was strongly affected by temperature and pH. The thermal stability of enzymes is one of the most important application criteria for different applications. After immobilization, the temperature curve of TtTreS showed a similar pattern to that of free enzyme (Figure 3a). The activity of TtTreS significantly increased with increasing reaction temperature until 50 °C, and then declined with further increasing reaction temperature. In the temperature range of 40 °C to 50 °C, immobilized TtTres presented higher relative activity than free TtTres. Both free and immobilized TtTreS reached maximum relative activity at the optimal temperature of 50 °C. However, immobilized TtTreS showed higher relative activity than free TtTreS in the temperature range of 50 °C–70 °C (Figure 3a), indicating that the thermal stability of TtTreS was improved by immobilization. This may be attributable to immobilization helping to prevent the multimeric TtTres from dissociating. Immobilization also provides TtTreS with potential application in a relatively wide range of temperatures from 40 °C to 70 °C.

The pH curve showed that immobilized TtTreS performed better than free enzyme at pH values between 4 and 8 (Figure 3b). Immobilized and free TtTreS reached the maximum relative activity at pH 8 and pH 9, respectively. The optimal pH of TtTreS was shifted to the neutral region after immobilization, which was in agreement with the results of tannase immobilization [30]. In addition, the pH change of immobilized and free enzyme during catalytic reaction was determined (Table 2). The pH decreased during trehalose conversion by TtTreS. In the present study, immobilized TtTreS exhibited a change of pH (∆pH 0.7), while that of free TtTreS was ∆pH 2.5. This result contributed to the stability and activity maintenance of immobilized TtTreS. The results showed that pH change affected the activity of enzymes. In our case, free enzyme was affected more than immobilized enzyme by pH change. The isoelectric point (pI) of the enzyme is important as it affects the activity and stability of the enzyme. Immobilization may affect the pI of enzymes. When the pH changes, the free enzyme may tend to form aggregates in solution. Meanwhile the immobilized enzyme was bonded to the material which decreased the formation of aggregates.

### 2.3. Reusability of Immobilized TtTreS

**Figure 4:**
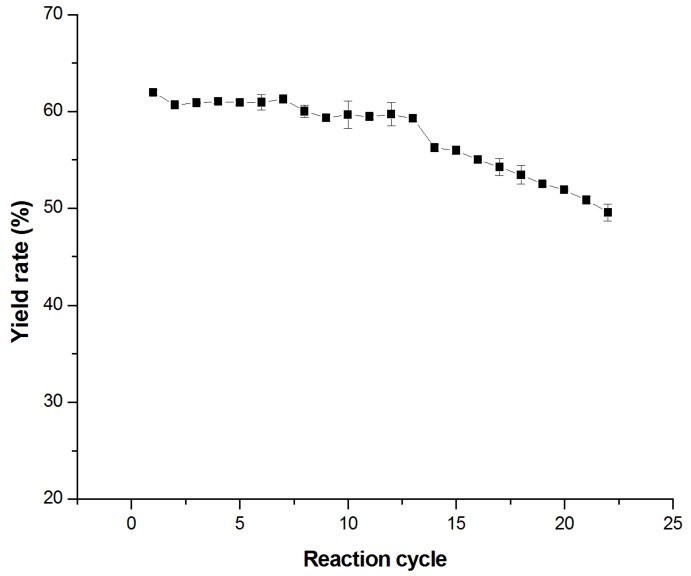
The reaction cycles of immobilized TtTreS. The reusability of immobilized TtTreS was evaluated by consecutive trehalose production cycles; 1 h represents 1 cycle. The data represent the mean values and standard deviations of three independent experiments.

The reusability of immobilized enzyme is one of the most important parameters for industrial application in the future. In the present study, the reusability of TtTreS was investigated by batch reactions for 22 cycles. After each enzymatic reaction, immobilized TtTreS was filtered and recovered by PBS and then reused in a new substrate solution of maltose. We determined the kinetic parameters of enzymes to compare the two kinds of enzymes (Table 3). The results showed that immobilization decreased some properties of the enzyme. Trehalose yield in TtTreS recycling is shown in Figure 4. The trehalose yield of immobilized TtTreS in first cycle reached 61.52%. The residual activity was slightly reduced with the increase of reuse cycles. After 22 cycles of enzymatic reaction, the immobilized TtTreS still retained 81% of its initial trehalose yield. The loss of activity may not only be attributed to leakage of the enzyme from the support, but also enzyme inactivation during recovery steps [31]. Meanwhile, enzyme will slowly lose activity with time in the bioconversion because of pH, temperature, and other elements. For industrial production, the cost of the bioreaction is an important factor to be considered.

Researchers have constructed an integrated biocatalytic process for trehalose conversion involving whole cells and separation. This strategy produced 0.675 g trehalose when 1 g maltose was consumed as substrate at 30 °C within 80.5 h [32]. Another report used immobilized trehalose synthase from *Picrophilus torridus* for trehalose production. The conversion yield of trehalose was 64.1% within 6 h and the enzyme retained 80% of initial yield after 24 cycles [21]. Compared to these works, our study suggested that immobilized TtTreS exhibits both promising reusability of enzymes and remarkable trehalose conversion yield.

## 3. Materials and Methods

### 3.1. Bacterial Strain and Medium

*E. coli* BL21(DE3) harboring pET-22b-tttreS and pCS27-sigma-32-GroeL-GroeS-DnaK-DnaJ was constructed in our previous study [10]. Luria–Bertani (LB) medium and Terrific Broth (TB) were used for pre-cultures in shake flasks and fermentation cultures in a 2.5-L fermenter (INFORS HT minifors, Switzerland), respectively. Luria–Bertani (LB) medium for pre-cultures in test tubes and shake flasks contains 10 g tryptone L^−1^, 5 g yeast L^−1^ extract and 10 g NaCl L^−1^. Terrific Broth (TB) medium for cultures in 2.5-L fermentation tanks contains 12 g tryptone L^−1^, 24 g yeast extract L^−1^, 12.54 g K_2_HPO_4_ L^−1^, 2.31 g KH_2_PO_4_ L^−1^, and 4 mL glycerol L^−1^. When needed, ampicillin and kanamycin were added to the medium at 100 μg/mL and 50 μg/mL, respectively. Lactose was added to a final concentration of 1.5 mM to induce protein expression. Supplemental medium contained 200 g glycerol L^−1^ and 25 g MgSO_4_·7H_2_O L^−1^.

### 3.2. Expression and Prepararion of TtTreS

After overnight culture at 37 °C and 200 rpm, 10% (*v*/*v*) seed culture was inoculated into 100 mL LB medium and cultured for another 12 h to reach an OD_600_ of 4~5 as the secondary seed. Then, 10% (*v*/*v*) secondary seed culture was inoculated into a 2.5-L fermenter containing 1 L autoclaved TB medium to start the fed-batch fermentation. At the first stage (3 h), we kept the culture at 37 °C and DO state at 20% by regulating the stir speed and the aeration rate (maximal rate is 1.5 VVM). At the second stage (induction stage), the culture was cooled to 28 °C when OD_600_ of the culture reached 10, then lactose was added to induce protein expression. When there was a sudden increase in DO value, the fed-batch cultivation was started by feeding supplemental medium at the speed of 1.2 g L^−1^ h^−1^ until the biomass reached its constant value.

The cultivation broth was centrifuged at 15,000× *g* for 5 min at 4 °C. The cell pellets were collected and washed twice in 50 mM PBS (pH 7.0). Then, the bacterial cells were broken using an ultrasonic cell disruptor to release the intracellular enzyme. After centrifugation, the supernatants were used as crude enzyme for enzyme immobilization.

### 3.3. Synthesis of Support Materials and TtTreS Immobilization

The process of enzyme immobilization is shown in Figure 1. Silicalite-1 was firstly activated by 0.1 mM NaOH for 10 min. Then, the activated silicalite-1 was modified by 3-Aminopropyltrimethoxysillane (APS, 98% *w*/*v*) in toluene to provide an amino group on the surface of the material. Then, glutaraldehyde (GA, 0.25% *w*/*v*) was used as crosslinker to react with the amino groups of APS, providing aldehyde groups on the surface of the material to react with the amino groups of TtTreS. The resultant material, GA-APS-silicalite-1, was washed with water. Scanning electron microscopy (SEM) (HITACHI S-4700, Tokyo, Japan) was used to observe the surface of silicalite-1, APS-silicalite-1, and GA-APS-silicalite-1. TtTreS was immobilized by adding crude enzyme (20 mg/mL) to the supporting materials in PBS (50 mM, pH 7.0) solution at the ratio of 20 U/g and stirring at 200 rpm for 6 h at 24 °C. When the amount of enzyme loading ranged from 0.5 mL to 2.5 mL, it was found that 1.0 mL was the best choice. The immobilized TtTreS was washed with PBS for three times until the complete removal of unbound enzymes.

### 3.4. Effect of Temperature and pH on the Activity of Free and Immobilized TtTreS

The effect of temperature and pH on the activities of free and immobilized enzymes was evaluated in 5 mL of 20% (*w*/*v*) maltose solution containing 0.2 g immobilized TtTres with the speed of 180 rpm, respectively. When temperature was the variable, the pH was kept at 8.0. When pH was the variable, the temperature remained at 50 °C. Image J grayscale scanning was used to determine the protein amount. Enzyme activity was assayed using the protocol we reported previously, using 20% (*w*/*v*) maltose solution as substrate (50 mM PBS, pH 7.0) [11]. The production of trehalose was determined by HPLC after 10 min. The enzyme activity at optimal conditions was taken as 100% activity. The relative activity was calculated by the following equation:

$$\mathrm{Relative}\;\mathrm{activity}\;\left(100\%\right)=\frac{\mathrm{Activity}}{\mathrm{Maximum}\;\mathrm{activity}}\times 100$$

### 3.5. Determination of Trehalose, Maltose, and Glucose

The analysis of trehalose, maltose, and glucose was performed by HPLC (Hitachi, Tokyo, Japan) [10]. They were separated by a reverse-phase Venusil PS NH column (Agela) at 25 °C and detected by a refractive index detector. 87% Acetonitrile/13% water was used as mobile phase at a flow rate of 1 mL/min.

### 3.6. Trehalose Production by Immobilized TtTreS

The reusability of immobilized TtTreS was evaluated by repeated trehalose production using TreS-GA-APS-silicalite-1 at optimal conditions for 22 batches in 2% (*w*/*v*) maltose solution at 50 °C. Each reaction cycle lasted for 1 h. After each enzymatic conversation from maltose to trehalose, TtTreS-GA-APS-silicalite-1 was recovered by centrifugation and rinsed with PBS.

## 4. Conclusions

In this study, in order to produce trehalose on a large scale, surface modification of silicalite-1 was performed to immobilize crude trehalose synthase from *Thermus thermophilus* HB27 (TtTreS) to improve its reusability. The activity and stability of TtTreS against pH and temperature was greatly improved after enzyme immobilization. Enzyme immobilization also led to a decrease in the concentration of byproduct glucose from 22 g/L to 15 g/L, which could reduce the byproduct inhibition of TtTreS. Immobilized TtTreS retained 81% of its original activity after 22 reaction cycles in maltose solution. The excellent reusability demonstrated that immobilized TtTreS is promising for industrial applications.

## Figures and Tables

**Table 1:** Trehalose yield of first two recycling batches using slilicalite-1, APS-silicalite-1, and GA-APS-silicalite-1 as support.

Material	Silicalite-1	APS-Silicalite-1	GA-APS-Silicalite-1
1st batch	61.20%	35.09%	61.52%
2nd batch	0.00%	0.00%	60.28%

**Table 2:** The pH value and pH variation of free TtTreS and immobilized TtTreS.

Type of Enzyme	Optimal Reaction pH	pH at 0 h	pH at 24 h	∆pH
Immobilized-TtTreS	8.00	8.04 ± 0.12	7.30 ± 0.21	0.70 ± 0.09
Free-TtTreS	9.00	9.03 ± 0.08	6.50 ± 0.15	2.50 ± 0.07

**Table 3:** The K_m_ value and V_max_ of immobilized and free TtTreS.

Enzyme Type	K_m_ * Value (mM)	V_max_ * (µmol/min)
Immobilized TtTres	53.33 ± 5.21	2.93 ± 0.22
Free TtTres	14.12 ± 1.84	8.72 ± 0.38

* The data represent the mean values and standard deviations of three independent experiments.
